# The Wide World of Ribosomally Encoded Bacterial Peptides

**DOI:** 10.1371/journal.ppat.1004221

**Published:** 2014-07-31

**Authors:** Rebecca A. Flaherty, Stefan D. Freed, Shaun W. Lee

**Affiliations:** 1 Department of Biological Sciences, University of Notre Dame, Notre Dame, Indiana, United States of America; 2 Eck Institute for Global Health, University of Notre Dame, Notre Dame, Indiana, United States of America; 3 Center for Rare and Neglected Diseases, University of Notre Dame, Notre Dame, Indiana, United States of America; University of North Carolina at Chapel Hill School of Medicine, United States of America

## Bacterial Peptides: Enormous Diversity

Peptides are defined as short chains of amino acids that are linked by peptide bonds. In eukaryotes, peptides encompass an enormous range of structure and function, from signaling hormones, to anti-pathogen molecules, to powerful toxins. In bacteria, ribosomally produced peptides known as bacteriocins have been historically investigated for their potential antimicrobial activities [1]. However, recent efforts in genomics and natural product discovery have led to a tremendous “explosion” in the sheer number and diversity of ribosomally produced peptides from the prokaryotic domain. It has been estimated that all bacteria and archaea produce at least one, but more likely multiple, bacteriocin-like peptides that have a wide range of functions including antimicrobial toxins, virulence factors, and bacterial “hormones” that allow bacterial communities to organize multicellular behaviors such as biofilm formation. This article provides an overview of ribosomally produced bacterial peptides and their diverse roles in bacterial lifestyles, along with future prospects and recent computational and bioinformatic approaches aimed at decoding the overall “language” of these bacterially produced peptides.

## Structure and Classification of Small Bacterial Peptides

**Figure 1:**
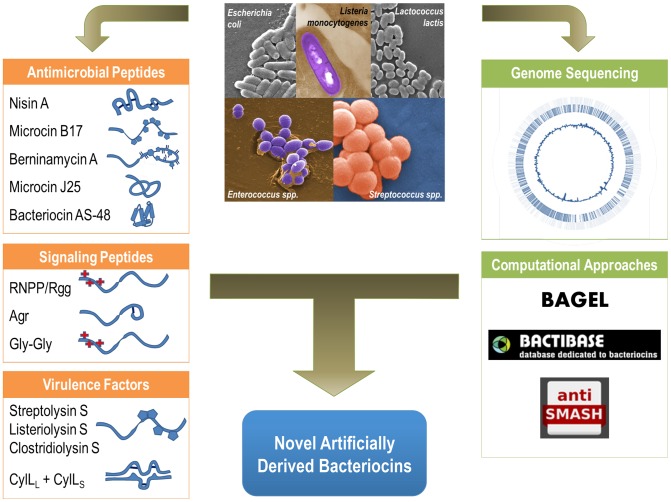
Functional diversity of ribosomally produced bacterial peptides. Bacterial peptides produced by both gram-positive and gram-negative bacteria include antimicrobial peptides such as Nisin and Microcin B17, known host virulence factors such as the Streptolysin S-like cytolysins, and the peptide cytolysin from *E. faecalis*. Bacterial peptides that structurally resemble bacteriocins are also utilized as signaling molecules. Computational and genomic approaches, such as BAGEL, BACTIBASE, and Anti-SMASH, can be combined with genomic data to catalog and discover new ribosomally produced bacterial peptides. Combining computational approaches with experimental data can guide the development of novel antimicrobials and artificially derived peptides with specific functions and targets.

Ribosomally produced bacterial peptides are a large class of compounds that encompass an extraordinary amount of chemical, structural, and functional diversity (Figure 1) [2], [3]. These small peptides can range from unmodified linear forms to highly modified, and sometimes circularized, structures. These modifications serve to confer specific chemical properties that could not be obtained via peptide synthesis alone, further increasing the number and complexity of these bacterial peptide families. Furthermore, certain modifications are thought to serve as an important safety mechanism to regulate the toxic activities of the bacterial peptide, thereby providing a level of control and self-immunity [2], [4]. Some of the major chemical classifications of ribosomally produced bacterial peptides include Lantibiotics such as Nisin, Linear azo(line)-containing peptides such as Microcin B17, Lasso peptides such as the *Escherichia coli* antibacterial peptide Microcin J25, and many others that continue to be discovered at a rapid pace [5]. Approaches to systematically classify all known ribosomally produced bacterial peptides have involved dividing groups based on: (1) particular prolific producers such as lactic acid bacteria, (2) particular modifications of the bacterial peptide, or (3) specific peptide activities. Indeed, given the sheer number and diversity of these bacterially produced compounds, there is tremendous potential in the discovery and development of these natural products as therapeutics. It has been noted that with respect to bacteriocins, bacteria have, in essence, “already designed what clinicians and pharmaceutical industries are once again struggling to obtain” [2].

## Bacterial Peptides as Antimicrobial Compounds

Many bacterial peptides have been known to exhibit potent antimicrobial activity against competitor species of the producing microorganism. Nisin, whose activity was first reported in 1928 [6], has been used widely in the food industry to prevent food-borne pathogens [7]. The mechanism of Nisin's action is to bind to an intermediate in bacterial cell wall synthesis, resulting in bacterial killing through pore formation [8]. Nisin belongs to a larger group of bacteriocins known as lantibiotics; these compounds have been highly studied, given their ability to specifically target clinically important pathogens such as *Streptococcus pneumoniae*, Methicillin-resistant strains of *Staphylococcus aureus* (MRSA), and Vancomycin-resistant enterococci (VRE) [9]. Microcin B17, a linear peptide produced by particular strains of *E. coli*, is one of many bacteriocins that are enzymatically modified by the addition of heterocyclic residues [10]. Microcin B17 targets susceptible bacteria by inhibiting bacterial DNA gyrase [11]. In contrast to Nisin, whose antimicrobial activity is most active against gram-positive bacteria, Microcin B17 has been shown to be effective against a wide range of gram-negative pathogens, such as *E. coli*, *Salmonella*, *Shigella*, and *Yersinia* species. A widely studied example of an unmodified bacterial peptide is the enterococcal bacteriocin AS-48, which has antimicrobial activity against gram-positive pathogens such as *Listeria monocytogenes* [12]. AS-48 bacteriocin belongs to a larger class of unmodified peptides that adopt a particular native structure that is critical for their activity.

## Bacterial Peptides as Virulence Factors

Recent discoveries have uncovered the fact that long-known potent bacterial toxins such as Streptolysin S (SLS) are actually small, ribosomally produced peptides, whose enzymatic modifications are highly similar to those of known bacteriocins, such as Microcin B17. In addition to their well-described role in microbial warfare against competing bacterial species, these peptide toxins are beginning to be recognized as key contributors to initiating host disease. Streptolysin S has been identified as a major contributing factor in successful translocation of *Streptococcus pyogenes* across the epithelial barrier through a mechanism involving the disruption of intracellular junctions via cleavage of occludin and E-cadherin [13]. The ability of peptide toxins such as SLS to prevent phagocytic clearance can also be mediated through direct killing of immune cells. A series of simple in vitro experiments exploring the effects of SLS on mouse peritoneal macrophages in the early 1970s provided the first indication that bacteriocin-like toxins can exhibit leukotoxic effects [14]. Like *S. pyogenes*, *Enterococcus faecalis* also produces a peptide cytolysin (encoded by the *cyIL* gene cluster) that is capable of lysing neutrophils and macrophages to avoid immune clearance [15]. Interestingly, several microbial peptide toxins have also been shown to have synergistic activity with other bacterial virulence factors, suggesting that, in fact, these bacterial peptides may serve the dual role of causing direct damage to the host while also increasing the overall virulence output. For example, Hung et al. utilized a murine infection model to demonstrate that the peptide toxin SLS synergizes with the unrelated streptococcal pyrogenic exotoxin B (SpeB) during infection to enhance several features of pathogenesis, including inhibition of phagocytic clearance and the induction of macrophage apoptosis [16]. In commensal bacteria such as *Lactobacillus plantarum*, it has been shown that production of antimicrobial bacteriocins can modulate the immune response of dendritic and peripheral blood mononuclear cells as well as alter host cytokine profiles versus nonbacteriocin producing mutants [17].

## Bacterial Peptides as Communication Signals

Many gram-positive bacteria use small peptides to communicate within a multicellular community to regulate processes such as cellular density, biofilm formation, competence for mating, and coordinated control of virulence [18]. Quorum sensing, the act of bacterial communication via extracellular diffusible molecules, allows bacteria in many cases to synchronize group behavior and facilitate coordinated events. In gram-negative bacteria, N-acyl homoserine lactones have been extensively studied for their role as communication molecules; however, in gram-positive bacteria, recent studies have revealed that small peptides function as the predominant signaling molecule of choice. One of the earliest discoveries of peptide pheromone signaling is the Agr system in *Staphylococcus aureus*, which uses a small cyclic peptide, known as autoinducing peptide (AIP), to communicate in a multicellular setting to control the expression of virulence genes for a coordinated effect on host pathogenesis [19]. Additionally, three other major groups of bacterial peptides have been studied for their roles in intercellular communication, which are the Gly-Gly processed peptides, the RNPP systems peptides, and the Rgg motif signaling peptides [20]. In some cases, reports are emerging that these peptides control multiple behaviors in bacteria. In *Streptococcus intermedius*, the quorum signal peptide known as competence stimulating peptide (CSP) was found not only to be involved in regulating competence for natural transformation but also to regulate and promote biofilm formation [21]. Multifunctional roles for bacteriocins are becoming a wider and more accepted phenomenon. For example, reports have suggested that Nisin can act both as an antimicrobial molecule and through an autocrine signaling mechanism to potentiate Nisin production in a cell density–dependent manner [22].

## Understanding the “Grammar” of Bacterial Peptides

Genome mining has been an important technological resource in the discovery of novel natural products, such as bacteriocins. Bacteriocin-like peptides are highly attractive candidates for genome mining, as these natural products are genetically encoded with nearby genes encoding their corresponding modifying enzymes. Proximity to genes encoding known modifying enzymes can aid in the identification of peptide biosynthesis gene clusters [23]. In many cases, several metabolites have been identified from “cryptic” or “orphan gene clusters” [24]. These cryptic gene clusters have demonstrated that new, as-yet-uncharacterized enzymology is likely to be involved in the assembly of the final natural product, likely leading to greater diversities of bacterial peptides than have previously been appreciated.

Many web-based Bacteriocin gene mining and annotation tools have been developed to aid in the identification, characterization, and classification of novel bacteriocins. These include mining tools such as BAGEL (http://bagel.molgenrug.nl) and bacteriocin repositories such as BACTIBASE (http://bactibase.pfba-lab-tun.org/main.php). Anti-SMASH is a recently developed website that expands genome mining to not only bacteriocins but a host of other genetically identifiable antibiotics and other secondary metabolites [25]. Although these tools are rapidly expanding the repertoire of bacterially produced peptides, one elusive goal has been to deduce the function of a given bacterial peptide from the structural and genetic information alone. Recent computing approaches, however, have begun to address the goal of using purely in silico approaches in order to predict the specific activity of peptides and proteins. Loose et al. have postulated a “linguistic” model for the design of antimicrobial peptides. The repeated usage of particular amino acid sequences that are common to antimicrobial peptides led these investigators to propose a certain grammar that governs the “language” of antimicrobial peptides [26]. In a recent study, Gupta et al. used a database of toxic and nontoxic peptides coupled with machine learning and a quantitative matrix approach to predict whether a given peptide will have toxic properties [27]. Their program, *Toxipred*, functions to essentially predict the relative toxicity of a given peptide based on sequence alone. Although still relatively new, programs such as these and others, combined with the rapid pace of genomic discovery, will rapidly accelerate the pace of drug discovery in these bacterial peptide families.

## Conclusions

Ribosomally produced bacterial peptides have seen a recent surge in interest due to new structural, biochemical, and microbiological tools, along with advances in genome sequencing and bioinformatics. Computational algorithms and bacterial peptide databases are rapidly growing as more of these compounds are discovered and deposited. Although traditionally bacteriocins have been classified as having antimicrobial properties, recent findings suggest that bacteria utilize bacteriocin-like peptides to perform a myriad of roles, including intercellular communication, host colonization and manipulation, as well as the exciting finding that these small peptides can have multiple functions in a given microorganism. Computational-based approaches, coupled with experimental data, will allow investigators to decipher the overall “language” of these ribosomally produced bacterial peptides such that novel antimicrobials and artificially derived peptides with specific functions and targets can be soon developed—provided that we will be able to “speak its language.”

## References

[1] Cotter PD, Ross RP, Hill C (2013) Bacteriocins - a viable alternative to antibiotics? Nat Rev Microbiol 11: 95–105. 23268227 10.1038/nrmicro2937

[2] Baba T, Schneewind O (1998) Instruments of microbial warfare: bacteriocin synthesis, toxicity and immunity. Trends Microbiol 6: 66–71. 9507641 10.1016/S0966-842X(97)01196-7

[3] Sit CS, Vederas JC (2008) Approaches to the discovery of new antibacterial agents based on bacteriocins. Biochem Cell Biol 86: 116–123. 18443625 10.1139/O07-153

[4] Garrido MC, Herrero M, Kolter R, Moreno F (1988) The export of the DNA replication inhibitor Microcin B17 provides immunity for the host cell. Embo J 7: 1853–1862. 3049078 10.1002/j.1460-2075.1988.tb03018.x PMC457178

[5] Arnison PG, Bibb MJ, Bierbaum G, Bowers AA, Bugni TS, et al (2013) Ribosomally synthesized and post-translationally modified peptide natural products: overview and recommendations for a universal nomenclature. Nat Prod Rep 30: 108–160. 23165928 10.1039/c2np20085f PMC3954855

[6] Rogers LA (1928) The Inhibiting Effect of Streptococcus Lactis on Lactobacillus Bulgaricus. J Bacteriol 16: 321–325. 16559344 10.1128/jb.16.5.321-325.1928 PMC375033

[7] Lubelski J, Rink R, Khusainov R, Moll GN, Kuipers OP (2008) Biosynthesis, immunity, regulation, mode of action and engineering of the model lantibiotic nisin. Cell Mol Life Sci 65: 455–476. 17965835 10.1007/s00018-007-7171-2 PMC11131864

[8] Bierbaum G, Sahl HG (2009) Lantibiotics: mode of action, biosynthesis and bioengineering. Curr Pharm Biotechnol 10: 2–18. 19149587 10.2174/138920109787048616

[9] Piper C, Cotter PD, Ross RP, Hill C (2009) Discovery of medically significant lantibiotics. Curr Drug Discov Technol 6: 1–18. 19275538 10.2174/157016309787581075

[10] Walsh CT, Nolan EM (2008) Morphing peptide backbones into heterocycles. Proc Natl Acad Sci U S A 105: 5655–5656. 18398003 10.1073/pnas.0802300105 PMC2311349

[11] Yorgey P, Lee J, Kordel J, Vivas E, Warner P, et al (1994) Posttranslational modifications in microcin B17 define an additional class of DNA gyrase inhibitor. Proc Natl Acad Sci U S A 91: 4519–4523. 8183941 10.1073/pnas.91.10.4519 PMC43817

[12] Mendoza F, Maqueda M, Galvez A, Martinez-Bueno M, Valdivia E (1999) Antilisterial activity of peptide AS-48 and study of changes induced in the cell envelope properties of an AS-48-adapted strain of Listeria monocytogenes. Appl Environ Microbiol 65: 618–625. 9925591 10.1128/aem.65.2.618-625.1999 PMC91070

[13] Sumitomo T, Nakata M, Higashino M, Jin Y, Terao Y, et al (2011) Streptolysin S contributes to group A streptococcal translocation across an epithelial barrier. J Biol Chem 286: 2750–2761. 21084306 10.1074/jbc.M110.171504 PMC3024771

[14] Ofek I, Bergner-Rabinowitz S, Ginsburg I (1972) Oxygen-stable hemolysins of group A streptococci. 8. Leukotoxic and antiphagocytic effects of streptolysins S and O. Infect Immun 6: 459–464. 4634952 10.1128/iai.6.4.459-464.1972 PMC422559

[15] Miyazaki S, Ohno A, Kobayashi I, Uji T, Yamaguchi K, et al (1993) Cytotoxic effect of hemolytic culture supernatant from Enterococcus faecalis on mouse polymorphonuclear neutrophils and macrophages. Microbiol Immunol 37: 265–270. 8350769 10.1111/j.1348-0421.1993.tb03209.x

[16] Hung CH, Tsao N, Zeng YF, Lu SL, Chuan CN, et al (2012) Synergistic effects of streptolysin S and streptococcal pyrogenic exotoxin B on the mouse model of group A streptococcal infection. Med Microbiol Immunol 201: 357–369. 22610375 10.1007/s00430-012-0241-6

[17] van Hemert S, Meijerink M, Molenaar D, Bron PA, de Vos P, et al (2010) Identification of Lactobacillus plantarum genes modulating the cytokine response of human peripheral blood mononuclear cells. BMC Microbiol 10: 293. 21080958 10.1186/1471-2180-10-293 PMC3000848

[18] Lyon GJ, Novick RP (2004) Peptide signaling in Staphylococcus aureus and other Gram-positive bacteria. Peptides 25: 1389–1403. 15374643 10.1016/j.peptides.2003.11.026

[19] Kong KF, Vuong C, Otto M (2006) Staphylococcus quorum sensing in biofilm formation and infection. Int J Med Microbiol 296: 133–139. 16487744 10.1016/j.ijmm.2006.01.042

[20] Cook LC, Federle MJ (2013) Peptide pheromone signaling in Streptococcus and Enterococcus. FEMS Microbiol Rev 38: 473–492. 24118108 10.1111/1574-6976.12046 PMC4103628

[21] Petersen FC, Fimland G, Scheie AA (2006) Purification and functional studies of a potent modified quorum-sensing peptide and a two-peptide bacteriocin in Streptococcus mutans. Mol Microbiol 61: 1322–1334. 16925560 10.1111/j.1365-2958.2006.05312.x

[22] Kleerebezem M (2004) Quorum sensing control of lantibiotic production; nisin and subtilin autoregulate their own biosynthesis. Peptides 25: 1405–1414. 15374644 10.1016/j.peptides.2003.10.021

[23] Challis GL (2008) Mining microbial genomes for new natural products and biosynthetic pathways. Microbiology 154: 1555–1569. 18524911 10.1099/mic.0.2008/018523-0

[24] Gross H (2007) Strategies to unravel the function of orphan biosynthesis pathways: recent examples and future prospects. Appl Microbiol Biotechnol 75: 267–277. 17340107 10.1007/s00253-007-0900-5

[25] Blin K, Medema MH, Kazempour D, Fischbach MA, Breitling R, et al (2013) antiSMASH 2.0–a versatile platform for genome mining of secondary metabolite producers. Nucleic Acids Res 41: W204–212. 23737449 10.1093/nar/gkt449 PMC3692088

[26] Loose C, Jensen K, Rigoutsos I, Stephanopoulos G (2006) A linguistic model for the rational design of antimicrobial peptides. Nature 443: 867–869. 17051220 10.1038/nature05233

[27] Gupta S, Kapoor P, Chaudhary K, Gautam A, Kumar R, et al (2013) In silico approach for predicting toxicity of peptides and proteins. PLoS ONE 8: e73957. 24058508 10.1371/journal.pone.0073957 PMC3772798

